# An Artificial Intelligence-Enabled Pipeline for Medical Domain: Malaysian Breast Cancer Survivorship Cohort as a Case Study

**DOI:** 10.3390/diagnostics11081492

**Published:** 2021-08-18

**Authors:** Mogana Darshini Ganggayah, Sarinder Kaur Dhillon, Tania Islam, Foad Kalhor, Teh Chean Chiang, Elham Yousef Kalafi, Nur Aishah Taib

**Affiliations:** 1 Data Science & Bioinformatics Laboratory, Institute of Biological Sciences, Faculty of Science, Universiti Malaya, Kuala Lumpur 50603, Malaysia; mogana@ummc.edu.my (M.D.G.); foadkalhor91@gmail.com (F.K.); markcc95@gmail.com (T.C.C.); elham@um.edu.my (E.Y.K.); 2 Department of Surgery, Faculty of Medicine, Universiti Malaya, Kuala Lumpur 50603, Malaysia; tania.omee@um.edu.my

**Keywords:** artificial intelligence, automated analysis, breast cancer, machine learning, medical domain

## Abstract

Automated artificial intelligence (AI) systems enable the integration of different types of data from various sources for clinical decision-making. The aim of this study is to propose a pipeline to develop a fully automated clinician-friendly AI-enabled database platform for breast cancer survival prediction. A case study of breast cancer survival cohort from the University Malaya Medical Centre was used to develop and evaluate the pipeline. A relational database and a fully automated system were developed by integrating the database with analytical modules (machine learning, automated scoring for quality of life, and interactive visualization). The developed pipeline, *i*Survive has helped in enhancing data management as well as to visualize important prognostic variables and survival rates. The embedded automated scoring module demonstrated quality of life of patients whereas the interactive visualizations could be used by clinicians to facilitate communication with patients. The pipeline proposed in this study is a one-stop center to manage data, to automate analytics using machine learning, to automate scoring and to produce explainable interactive visuals to enhance clinician-patient communication along the survivorship period to modify behaviours that relate to prognosis. The pipeline proposed can be modelled on any disease not limited to breast cancer.

## 1. Introduction

According to the 2020 GLOBOCAN estimates of cancer incidence and mortality, cancer is the first or second leading cause of death in 112 of 183 countries and ranks third or fourth in other 23 countries (Bray et al., 2020). Female breast cancer has surpassed lung cancer as the most commonly diagnosed cancer, with an estimated 2.3 million new cases in 2020 [1]. Breast cancer still remains the most common cancer in women worldwide [2].

Over the past two decades, the incidence of breast cancer has continued to escalate in most Asian countries [3,4]. The mortality rate of breast cancer is higher in developing countries despite the number of cases being lower compared to developed countries [5]. In Malaysia, 50–60% of breast cancer cases are detected at late stages, and hence the survival of the patients is one of the lowest in the region [6,7,8]. Survival of patients is also dependent on the ethnicity of patients, other than treatments and stage at presentation [9]. We need to explore other predictive factors such as Body Mass Index (BMI) and co-morbidities in building AI pipelines that could assist in clinical decision making and survivorship recommendations.

The performance of artificial intelligence (AI) in healthcare has been very promising to date [3]. However, the advancement in hospital-based healthcare is limited to free text entries and digitization of paper-based questionnaires. The idea of engaging databases, automated data analytics and machine learning is still in the budding stage, where clinicians rarely experience the benefit of these systems [3,4].

The difficulties faced by clinicians to make use of AI-enabled systems are manifold. One of the main factors is the adaptation to the technological environment. This is due to the complicated structure of machine learning algorithms, which minimizes the understanding and reliability in the healthcare domain [3]. Physicians are not convinced by the black box theories offered by machine learning algorithms, as they prefer to understand how the available systems produce automatic decisions and recommendations [5]. On the other hand, the limitation of cost-effective and open-source systems makes AI less preferred.

Data-driven research is paramount for clinical decision-making [10], but AI-based data-driven systems are sometimes not easily interpretable [5]. Hence, improved interactive visualization techniques and automated visual analytics are required to facilitate the interpretation to help clinicians in decision making [11]. Besides, clinicians also face problems in managing data from data collection and data storage to data-driven automated analytics. Most of the hospital based clinical studies were predominantly performed using conventional statistics [5,6,7,8,12,13], and the datasets used for prediction analytics were from different sources such as spreadsheets or were semi-automated (from preloaded databases), which promote errors in conventional statistics [14,15,16,17]. This limitation is further intensified by the fact that these widely used datasets must be stored in third-party software to perform further analyses.

Undeniably, machine learning algorithms can provide exceptional results and decisions from premium training data based on their built-in functions automatically. Nevertheless, when dealing with large amounts of data, more hybrid models need to be designed to resolve the issues that arose in data science for knowledge extraction especially in healthcare [18]. Despite the dynamic nature of healthcare analytics, automated machine learning models for variable selection and survival analysis for cancer with interactive visualizations are still not available in existing tools or websites. This is the major limiting factor for clinical decision-making.

The aim of this study is to propose a pipeline to develop a fully automated clinician-friendly AI-enabled platform for breast cancer survival prediction. In order to demonstrate the pipeline, we developed *i*Survive, a fully automated platform, which incorporates digitized questionnaires for data collection, database for data storage and management, automated machine learning analytics modules for survival prediction, and automated quality of life scoring and explainable interactive visualizations for clinician-patient communication during clinic consultations along the survivorship period to modify behaviors that relate to prognosis to improve the care of their patients.

## 2. Materials and Methods

**Figure 1:**
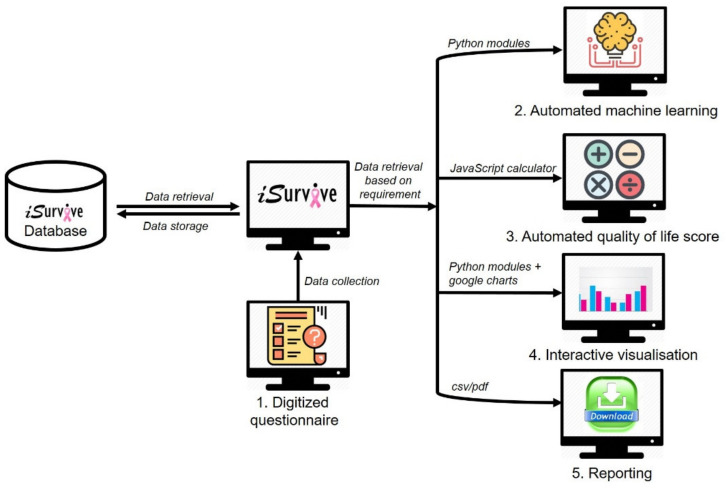
*i*Survive development workflow.

The methods used to develop *i*Survive (Figure 1) are explained in detail.

### 2.1. Database Design

The dataset consists of 1000 patients′ records and 633 variables at five timelines, which are baseline, six months, one year, three years and five years from February 2012 until February 2019 [9]. The participants provided written-informed consent forms before being recruited for the study. These data were collected via paper-based questionnaires on socioeconomic, body composition, lifestyle (nutrition, physical activity), mental and socio-cultural condition, overall survival, and quality of life related factors of the patients, whereas clinical details were obtained from the hospital′s clinical registry. A database was developed combining both the clinical and lifestyle information of the patients using MySQL relational database management system (RDBMS) in XAMPP, version 7.3.24 (phpMyAdmin), platform, discovered by Apache Friends. The relational algebra used to extract the data of the same patients from different datasets is represented in a Cartesian product (Algorithm 1).
**Algorithm 1:** Cartesian product to select lifestyle and clinical factors from different tables.1:Select 13 lifestyle factors, life status and survival years from table, mybcc and four clinical factors from table, clinical where the mybcc.RN = clinical.RN (select same patient ID from both tables)2:r = **σ**_mybcc.RN = clinical.RN_ ((π_RN,l1,l2,l3…,l13, lifestatus, survivalyears_ (mybcc)) × (π_RN,c1,c2,c3.c4_ (clinical)3:Definition:4:r                                = relational database5:**σ**                               = selection6:Π                               = projection7:×         = Cartesian product8:mybcc        = data table, which contains lifestyle factors9:clinical       = data table, which contains clinical factors10:RN         = patient ID/primary key in both tables11:lifestatus                = life status of the patients (Alive/Dead)12:survivalyears        = Overall survival years of the patients13:l1,l2,l3…l13           = 13 lifestyle factors14:c1,c2,c3,c4 = 4 clinical factors

### 2.2. Pipeline to Develop iSurvive

The modules in *i*Survive are digitized questionnaires, automated machine learning, quality of life scoring, interactive visualizations, and data download for secondary storage. HTML5 and CSS version 2.1 were used to design the whole user interface, whereas the development of analytics modules using PHP version 8.0, Python version 3.8, and JavaScript 12th edition are explained in detail.

#### 2.2.1. Digitization of Questionnaires

633 questions [9] for each timeline (baseline, six months, one year, three years and five years) were digitized in HTML forms via MySQL-PHP database connection. The digitized questionnaire consists of features such as text fields, radio buttons, check boxes, and date fields. The digitized questionnaires are designed to search for patients based on appointments, register new patients, collect data, and update data from time to time.

#### 2.2.2. Automated Machine Learning Module

**Figure 2:**
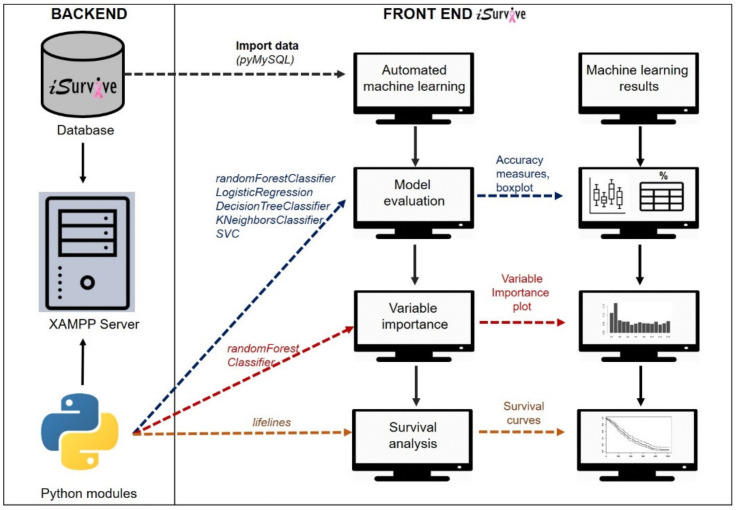
Automated machine learning in *i*Survive.

One of the core features of *i*Survive is the automated machine learning module, which includes three steps: (i) model evaluation, (ii) variable importance, and (iii) survival analysis as shown in Figure 2.

The interface was designed using HTML where the required Python modules were embedded in XAMPP server using cgitb Python module, which executes Python scripts to connect to the database, fetch data, perform analytics, and display the results on *i*Survive interface as shown in Algorithm 2.
**Algorithm 2:** Python-HTML integration for automated machine learning.1:a = query1 + ((pm_1_,pm_2_,…,pm_n_) + ps + ph)2:Definition:3:a       = automated analysis4:query1     = **σ**_mybcc.*RN* = clinical.*RN*_ ((π*_RN,l1,l2,l3…,l14,lifestatus,survivalyears_* (mybcc)) × (π*_RN,c1,c2,c3.c4_* (clinical)  (Refer to Algorithm 1)5:pm_1_,pm_2_,…pm_n_ = Pyhton modules6:ps       = Python script to run each analysis7:ph       = Python-HTML connection via *cgitb*

As the initial step, the dataset from MySQL database was imported using *pymysql* Python module (refer to Algorithm 1). Then, the data were partitioned into target and independent variables. Model evaluation was performed using five different algorithms (RandomForestClassifier, LogisticRegression, DecisionTreeClassifier, KNeighborsClassifier, and SVC). The dataset (*n* = 1000) was split into training (70%) and testing (30%) sets. The five different models were evaluated using different measures which are sensitivity, specificity, area under the receiver operating curve (AUC) and accuracy. The best model was selected based on the accuracy measure (%), visualized as a bar chart on *i*Survive.

Variable importance was performed using RandomForestClassifier with four clinical factors (stage, estrogen receptor (ER) status, progesterone receptor (PR) status, CERB2 status), 13 lifestyle factors (age, recurrence, occupation, marital status, ethnicity, income, education, body mass index (BMI), menarche, menopausal, alcohol intake, smoking status and stress level) as independent variables and survival years as the target variable. The total number of trees (ntree) used was 500 and the total number of variables for each split was 4. The variable importance strategy was adopted from a previous study on machine learning analysis using breast cancer data [19].

Survival analysis was performed using lifelines Python module with the information of survival years and life status of the patients in the selected cohort using Kaplan–Meier, a non-parametric statistic that allows the estimation of the survival rate. The functions in lifelines module computed the Kaplan–Meier estimator for truncated and censored data. The survival curves showed the estimation of survival years of patients based on the selected independent variable where BMI was used as a sample to automate the survival analysis on *i*Survive.

#### 2.2.3. Automated Quality of Life Scoring

The automation of quality of life scoring was done following the European Organisation for Research and Treatment of Cancer (EORTC) manual [20]. It contains 53 questions (QLQ-C30 version 3.0 and Breast Cancer Module QLQ-BR23). The scoring for QLQ-C30 consists of three sections (Global health status, functional scales and symptom scales) whereas the Breast Cancer Module QLQ-BR23 consists of two sections, functional scales and symptom scales as shown in Table 1 and Table 2.

For all scales, the RawScore, RS is the mean of the component items, *n* is the number of questions, range is the difference between the possible maximum and the minimum response to individual questions.

RawScore:

RS = (I1 + I2 +...+ In)/n

Functional scales:

Score = 1 − (RS − 1) × 100

range

Symptom scales and Global health status/QoL:

Score = {(RS − 1) range} × 100

MySQL-PHP connection and JavaScript were used to extract data from the database that automatically calculates the scores based on predefined formulas. The outputs can then be saved in the database and can be visualized in the *i*Survive interface.

#### 2.2.4. Interactive Visualizations

Interactive visualizations were developed using Python and MySQL database connection to fetch data directly from the database to visualize the analytics on iSurvive interface. The data can be visualized in bar charts, line graphs, tables, and other explainable plots. Algorithm 3 below explains the process model of automated visualization from the database.
**Algorithm 3:** Model of the automated visualization from database. 1:v = query2 + (c_1_,c_2_,…,c_n_)2:Definition:3:v      = visualization4:query2   = select (variable1, variable2,..., n) from table1 where RN = $search5:c_1_,c_2_,…,c_n_ = different types of charts

#### 2.2.5. Download Module

The data stored in the database were made available for download in .xlsx and .csv formats while the interactive visualizations for a specific patient can be downloaded or saved in pdf format for reporting. The download module was developed using PHP-MySQL database connection.

## 3. Results

The results of the fully automated pipeline (features and usability) of iSurvive are presented in the order of digitized questionnaire, automated machine learning, automated quality of life scoring, interactive visualization and download module. The main menu contains “APPOINTMENTS”, “ANALYTICS”, “DOWNLOAD”, and “LOGOUT”.

### 3.1. Digitized Questionnaire in iSurvive

**Figure 3:**
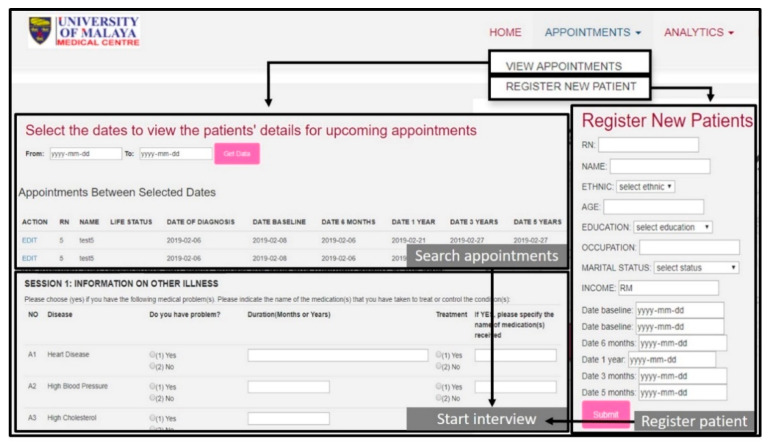
Digitized questionnaires in *i*Survive.

The ‘APPOINTMENTS′ menu navigates to the digitized questionnaires. It contains two submenus which are “VIEW APPOINTMENTS” and “REGISTER NEW PATIENT”. The view appointments link enables the user to search for patients between selected dates, and then enter their data based on the questionnaires. The register new patient link enables the user to register a newly recruited patient for this study by entering demographic details then continuing with the questionnaires. The usability of the digitized questionnaires is illustrated in Figure 3.

### 3.2. Automated Machine Learning in iSurvive

**Figure 4:**
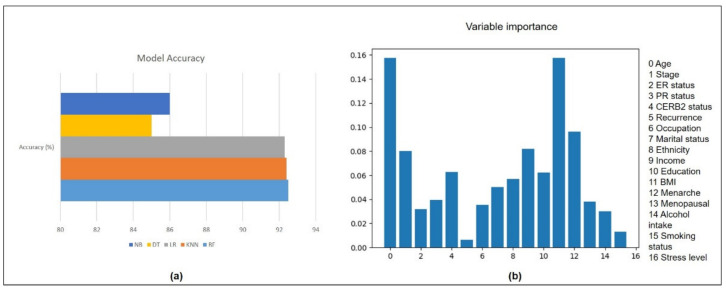
(**a**) Bar chart showing model accuracy measures of five algorithms (RF: 92.5%, KNN: 92.4%, LR: 92.3%, DT: 85.0%, NB: 86.0%). (**b**) The variable importance scores of 16 variables in ascending order (BMI: 0.91, Age: 0.15, Stage: 0.14, Income: 0.07, Menarche: 0.06, Marital status: 0.05, Ethnicity: 0.05, CERB2 status: 0.04, Education: 0.04, PR status: 0.03, Occupation: 0.02, ER status: 0.02, Menopausal: 0.02, Recurrence: 0.02, Alcohol intake: 0.01, Smoking status (0.01), Stress level: 0.00).

**Figure 5:**
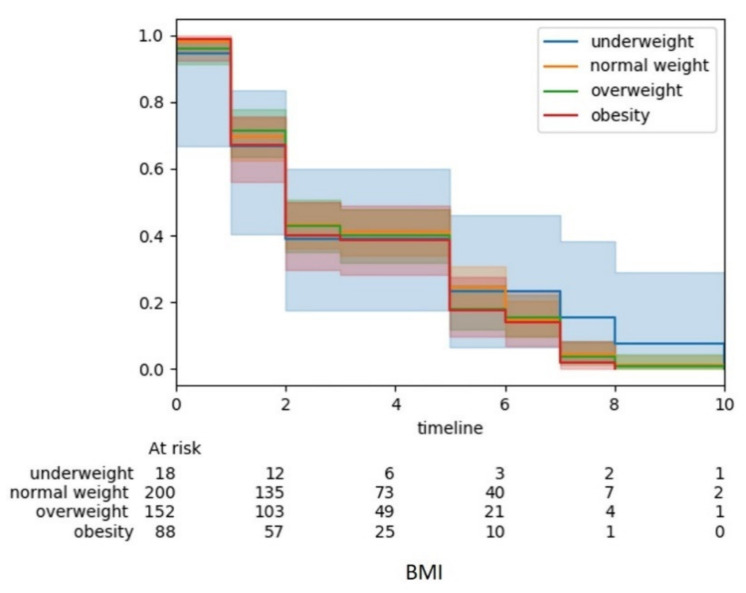
Survival curves using BMI and survival years.

Model evaluation using five different algorithms reported that the random forest is the best algorithm for this cohort with an accuracy of 92.5%. Variable importance using random forest reported that the five most important factors affecting the survival of breast cancer patients are BMI, age, stage, family income, and menarche. The survival curves were plotted using the variables BMI, survival years and life status (alive/dead). The model accuracy bar chart, variable importance plot and survival curve are shown in Figure 4 and Figure 5.

### 3.3. Automated Quality of Life Scoring in iSurvive

**Figure 6:**
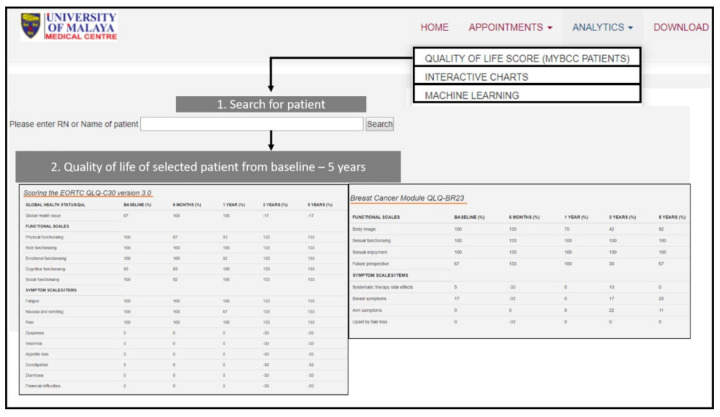
Quality-of-life scoring page of *i*Survive.

Quality of life scoring was programmed under the “ANALYTICS” menu. The quality-of-life scoring enables the user to search for patients using their name or record number (RN) then, can view the patient′s quality of life score from baseline, six months, one year, three years and five years in the same table. The quality-of-life scoring page is shown in Figure 6.

### 3.4. Interactive Visualizations

**Figure 7:**
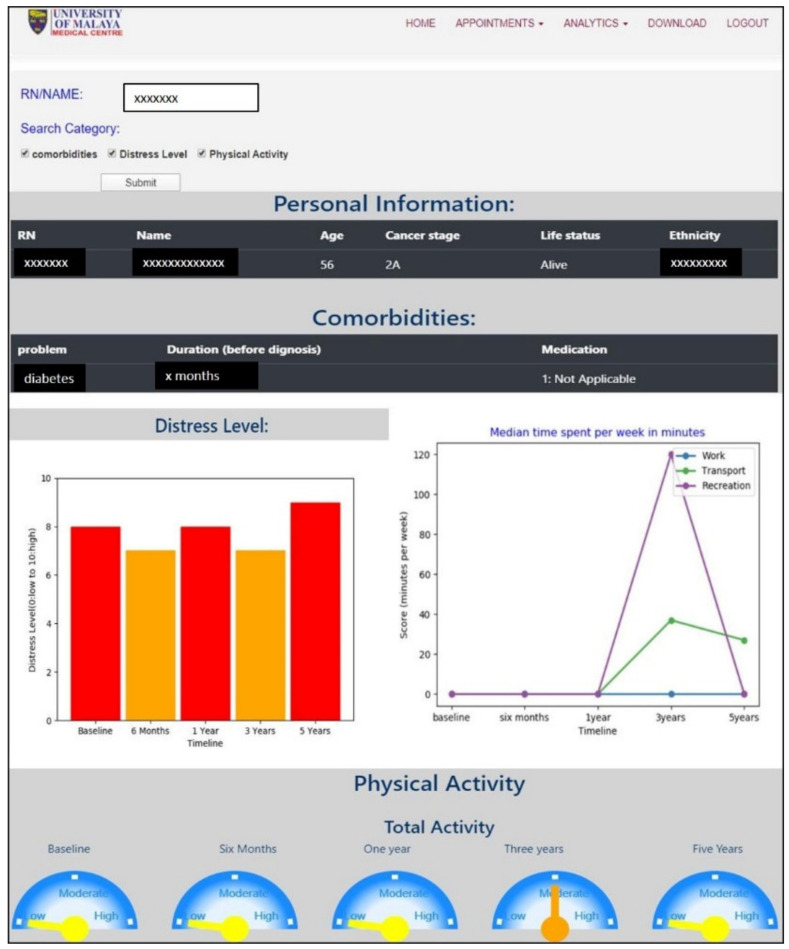
Interactive visualization showing the reports on personal information, comorbidity, physical activity measure, and distress level of a selected patient.

The interactive visualization module contains tables, bar charts, line graphs, and other explainable charts. The user can search for a specific patient′s RN, then filter the type of information to be viewed using thick boxes. The selected information will be fetched from different tables in the database and be displayed as a single report. An example of a patient report is shown in Figure 7.

### 3.5. Download Module in iSurvive

**Figure 8:**
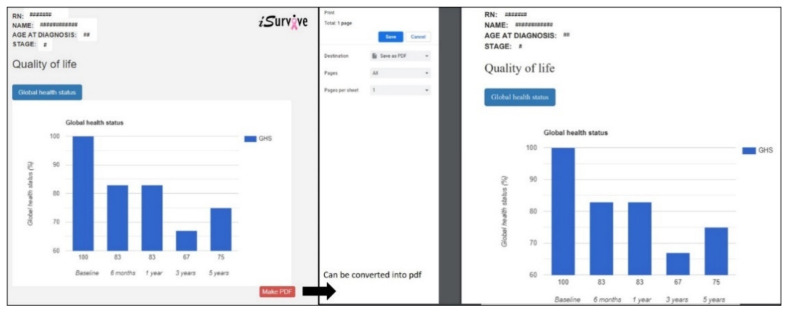
Quality-of-life chart, which can be exported to pdf from iSurvive.

A pdf sample of download is shown in Figure 8. The quality-of-life scoring for an individual patient is visualized in a column chart to compare the difference between follow up time after diagnosis. The individual patient report can be exported into pdf to provide the information to the patient while communicating with the clinician.

## 4. Discussion

### 4.1. Comparison with Previous Studies and Signifcance of This Study

In this study, we proposed a pipeline to develop a fully automated clinician-friendly AI-enabled platform for breast cancer survival prediction. We developed a feature-rich digital platform called *i*Survive which contains a database, digitized questionnaires, automated machine learning, automated quality of life scoring, interactive visualizations for clinician-patient communication, and a download module for data reporting. The digitized questionnaires have been developed for data collection, data update, and data management for further analytics whereas the database promotes a secure data storage system.

Automated analytics tool for machine learning analysis to perform model evaluation, variable importance, and survival analysis in *i*Survive has the potential to assist the clinicians as a decision support tool in cancer research. The model evaluation using five different algorithms reported closer accuracies with the highest score from random forest (92.5%). Random forest performed well in most clinical studies [19,21,22]. It has been reported to produce best accuracy and is superior to other techniques in terms of its ability in handling non-linear data and a large number of features [23]. Variable importance reported that the five most important factors affecting the survival of breast cancer patients are BMI, age, stage, overall family income, and menarche. Most of the prognostic breast cancer related studies are based on clinical data of the patients, while non-biomedical or lifestyle related research is limited [24]. Hence, lifestyle factors need to be included in breast cancer cohort studies where these factors are potentially modifiable to prolong survival [9,25].

The automated quality of life scoring in the *i*Survive serves as an advanced technology for researchers to analyze the changes in quality of life of the patients from the time of diagnosis up to five years follow up. Currently, paper-based questionnaires, which were developed by EORTC (Aaronson NK, et al., 1993) are being utilized by clinicians and researchers around the world to analyze the quality of life of cancer patients [26]. Even though digitization of the QoL module of EORTC has been done before [26], the scoring is still manual, where the clinicians need to organize the collected data from the digitized questionnaire to calculate the scoring manually. Similar types of paper-based cohort studies were updated only with digitized questionnaires for data collection, but not embedded with any analytical tool. A web-based patient reported outcomes system [6] was developed to collect data from patients using digital questionnaires, but the analysis was still done manually. Similarly, an online breast cancer risk assessment and risk management tool called iPrevent [27] was developed to collect breast cancer patients′ information on worry, anxiety and risk perceptions, in which data were evaluated using descriptive statistics. Another web and mobile-based invention for women treated for breast cancer to manage chronic pain and symptoms related to lymphedema was also developed for data collection with no embedded analytical tool within the digital system. A mobile breast cancer survivorship care app is also available to compare the performance of survivors from rural areas and urban areas [28]. However, these digital health platforms and apps are merely for data collection without any automated analytics. An innovation, iMOVE, a smartphone enabled health coaching intervention to promote long-term maintenance of physical activity in breast cancer survivors [29] was developed to provide personalized exercise program weekly for the participants. iMOVE is limited with one-sided benefit to the patients without any embedded analytical function on patient survival benefits which clinicians can utilize to provide motivation.

The interactive visuals in this novel pipeline empower the presentation of analyses where researchers can perform audits using the interface without going through the raw data again, which is not only cost effective but saves time. Additionally, this module helps clinicians to communicate with patients about their individual survival prediction and to motivate adherence to treatment and lifestyle interventions during the survivorship period. 

Managing the wealth of healthcare data helps enhance communication, patient care, development of personalized medicine, and clinical decision-making. Data is constantly being generated and stored in the form of electronic medical records (EMR) in healthcare organizations. Data science approaches can be applied to maintain and leverage this highly valuable healthcare data. Storage and management of data includes challenges such as data protection, data integration, data retrieval, and analytical software. In terms of data protection, paper-based forms are still being used to collect patient data. Manual data collection contributes to errors and missing values in data as well as time constraints. The database technology has evolved to replace papers or file based systems to digital systems, which now has become the best platform to store healthcare data to maintain seamless data integration, data analytics and data retrieval. Moreover, programs to encrypt or protect patient data are available as built-in in these database management systems. A data-warehousing approach enables creation of a platform that is integrated with analytical tools for the benefit of clinicians. In this study, the digitized questionnaires have been developed for data collection, data update, and data management for further analytics whereas the database promotes a secured data storage system. Automated analytical tools for machine learning analysis, are integrated into a database to perform model evaluation, variable importance, and survival analysis resulting in *i*Survive which has the potential to serve as a decision support tool in clinical practice.

Data visualization is streamlining the information into graphical outputs, which can be interpreted easily and quickly. Data visualization enables medical personnel to view the history of a single patient with a click of a button. If the same data visualization network is used in a healthcare organization, the data of the same patient from different departments can be integrated, retrieved from the database, and visualized based on requirements. The customization of visualizations includes type of graphs or charts, type of data (tables, images, test results, etc.) and specific information or variables related to a patient. Additionally, data visualization does not only help to improve response time but plays a pertinent role in presentation of results to patients, clinicians, policy makers, and the general public. Healthcare providers can make informed decisions by viewing the metrics from visualization and find ways to improve a patient′s outcome. Patient data is heterogeneous, with different formats, structures, and semantics. Most medical applications visualize patient data without integrating semantic information to structure the analysis, and hence it is a challenging task for clinicians to perform comparisons [30]. Integration of different databases to visualize the data of the same patients would be helpful for clinicians to compare results. The interactive visualizations in this novel pipeline *i*Survive empower the presentation of analyses where researchers can perform comparative audits using the interface without going through the raw data again, which is not only cost effective but saves a great amount of time. Additionally, this module helps clinicians to communicate with patients about their individual survival prediction and to motivate adherence to treatment and lifestyle interventions during the survivorship period. It has been found that primary doctors instilling healthy lifestyle messaging improves adherence to healthy lifestyle [31].

The machine learning approaches used in this study can be transformed into updated guidelines for academicians and researchers. Medical academic sector may use the methodologies demonstrated in this study for teaching and learning programs to educate medical students on the importance of machine learning. Moreover, researchers in the same field can follow the techniques and machine learning models explained in this study to conduct research and cohort studies not limited to breast cancer but any healthcare domain [19].

Clinical recommendations based on evidence need to be available and communicated effectively. The automated tools using machine learning algorithms help to augment patient care and to enhance clinician-patient communication as patients usually rely on the clinicians and hospitals for diagnosis, treatment, and follow up (especially those who are in critical conditions like cancer). Using the pipeline proposed in this study, clinicians can communicate the breast cancer treatment benefits and survival prediction with the patients, which ultimately promotes personalize care to individual patients to visualize the benefits of treatments. With such a facility, the patients will be able to decide on the best treatment to undergo based on the clinicians′ suggestions in order to improve their health. Additionally, the interactive visualization module in *i*Survive helps clinicians to communicate the information on lifestyle factors to improve lifestyle during survivorship period. 

### 4.2. Future Works and Recommendation

The software constraints we faced during the integration of machine learning modules with the *i*Survive platform are mainly on the programming languages. We integrated Python machine learning modules with the XAMPP platform, which enabled PHP-MySQL database connection to extract variables from the back-end relational database and to perform variable importance for survival. R, being a common software for machine learning, was not used in this study because it was not possible to establish a seamless integration in the *i*Survive XAMPP open-source cross platform web server development environment. In contrast, Python could be integrated with XAMPP to embed the machine learning solution in *i*Survive.

In any healthcare analysis, the number of missing values and quality of data is a pressing issue. While many efforts can be taken to minimize missing values and errors in data, this could not be solved completely. The *i*Survive back-end database was dependent on retrospective data, which had missing values due to the challenges faced in data collection especially for the five year follow-ups. Hence, maintaining the number of patients to complete the cohort and to have accurate analytical results was a notable shortcoming. In the next version of *i*Survive, other potentially modifiable factors to improve the survival of breast cancer patients will be analyzed using the machine learning enabled analytical tool.

*i*Survive will be validated by research assistants and clinicians by interacting with the patients for data collection and communicating personalized care through interactive visualizations. Internal validation will be performed by the researchers and clinicians in the UMMC, whereas external validation can be performed with the help of clinical experts, not limited to breast cancer, to obtain suggestions and ideas to improve the usability of the tool. Continuous improvements on the features and usability have been carried out through validation checks by users since its inception. Other potentially modifiable factors to improve the survival of breast cancer patients will be analyzed using the automated machine learning module. The *i*Survive pipeline can include other modules or sensors to collect longitudinal data from patients.

The proposed system serves as a one-stop center for clinicians to make data-driven decisions and recommendations for individual patients through automated machine learning and interactive visualizations. In the future, the pipeline used to develop *i*Survive can include other modules or sensors to collect longitudinal data from patients.

## 5. Conclusions

In this study, we proposed a pipeline to develop *i*Survive, a fully automated clinician-friendly AI-enabled platform for breast cancer survival analysis. It provides features such as digitized questionnaires, automated machine learning, automated scoring, and explainable interactive visualizations for clinician-patient communication. *i*Survive helps clinicians to communicate the information on lifestyle factors to improve lifestyle during the survivorship period. This development may serve as a motivation to use AI tools and systems in providing personalized patient care and survival, particularly for critical diseases like cancer.

## Figures and Tables

**Table 1:** Scoring the QLQ-C30 version 3.0.

	Scale	Number of Questions	Range	Questions Numbers
Global health status/QoL				
Global health status/QoL (revised) ^†^	QL2	2	6	29, 30
Functional scales				
Physical functioning (revised) ^†^	PF2	5	3	1 to 5
Role functioning (revised) ^†^	RF2	2	3	6, 7
Emotional functioning	EF	4	3	21 to 24
Cognitive functioning	CF	2	3	20, 25
Social functioning	SF	2	3	26, 27
Symptom scales/items				
Fatigue	FA	3	3	10, 12, 18
Nausea and vomiting	NV	2	3	14, 15
Pain	PA	2	3	9, 19
Dyspnoea	DY	1	3	8
Insomnia	SL	1	3	11
Appetite loss	AP	1	3	13
Constipation	CO	1	3	16
Diarrhoea	DI	1	3	17
Financial difficulties	FI	1	3	28

^†^ (Revised) scales are those that have been changed since version 1.0, and their short names are indicated in this manual by a suffix “2”—for example PF2.

**Table 2:** Scoring the Breast Cancer Module QLQ-BR23.

	Scale	Number of Questions	Range	Question Numbers
Functional scales				
Body image	BRBI	4	3	9–12
Sexual functioning ^†^	BRSEF	2	3	14, 15
Sexual enjoyment ^†^	BRSEE	1	3	16
Future perspective	BRFU	1	3	13
Symptom scales/items				
Systemic therapy side effects	BRST	7	3	1–4, 6, 7, 8
Breast symptoms	BRBS	4	3	20–23
Arm symptoms	BRAS	3	3	17, 18, 19
Upset by hair loss	BRHL	1	3	5

Questions for the scales marked “^†^” are scored positively.

## Data Availability

The de-identified data used in the current study (from the Malaysian Breast Cancer Survivorship Cohort (MyBCC), University Malaya Medical Centre) are not publicly available due to the Personal Data Protection Act 2010 (PDPA).

## Abbreviation

Artificial Intelligence	AI
Body Mass Index	BMI
European Organisation for Research and Treatment of Cancer	EORTC
Malaysian Breast Cancer Survivorship Cohort	MyBCC
Relational Database Management System	RDMS
Quality of Life	QoL
Structured Query Language	SQL
University Malaya Medical Centre	UMMC
